# Supplementary material for: Immunophenotypes Based on the Tumor Immune Microenvironment Allow for Unsupervised Penile Cancer Patient Stratification
Source: Cancers (Basel). 2020 Jul 4;12(7):1796. doi: 10.3390/cancers12071796 (PMC7407624; doi:10.3390/cancers12071796)
Supplement: Supplementary file 1 [file cancers-12-01796-s001.zip › cancers-858001-supplementary.docx]

Supplementary Materials: Immunophenotypes Based on the Tumor Immune Microenvironment Allow for Unsupervised Penile Cancer Patient Stratification

Chengbiao Chu, Kai Yao, Jiangli Lu, Yijun Zhang, Keming Chen, Jiabin Lu,
Chris Zhiyi Zhang and Yun Cao

**Table S1.** Tumor-microenvironmental characteristics associated with HPV status.

| **Continuous variable** | | | | | |
| --- | --- | --- | --- | --- | --- |
| **Variable** | **Total, N=** | **hrHPV^-^ (Mean ± SD)** | **hrHPV^+^ (Mean ± SD)** | ***P* ^a^** |  |
| **CD8s** | 177 | 5.56±1.54 | 5.68±1.28 | 0.602 |  |
| **CD8t** | 177 | 2.92±1.79 | 2.10±1.69 | 0.516 |  |
| **GrBs** | 176 | 5.61±1.00 | 5.92±0.95 | **0.049** |  |
| **GrBt** | 176 | 4.59±1.11 | 4.79±1.04 | 0.261 |  |
| **FOXP3s** | 177 | 5.10±1.26 | 5.04±1.16 | 0.769 |  |
| **FOXP3t** | 177 | 3.24±1.38 | 3.27±1.17 | 0.910 |  |
| **CD68s** | 177 | 7.61±0.36 | 7.46±1.05 | 0.169 |  |
| **CD68t** | 177 | 6.31±0.64 | 6.41±1.07 | 0.429 |  |
| **CD206s** | 175 | 5.33±1.40 | 5.17±1.51 | 0.525 |  |
| **CD206t** | 175 | 2.06±1.55 | 2.13±1.49 | 0.762 |  |
| **PD-1s** | 178 | 2.86±1.46 | 3.18±1.42 | 0.171 |  |
| **PD-1t** | 178 | 1.36±1.09 | 1.93±1.15 | **0.002** |  |
| **CTLA-4s** | 170 | 1.66±1.24 | 1.68±1.34 | 0.916 |  |
| **Categorical variable** | | | | | |
| **Variable** | **Total, N (%)** | **hrHPV^-^, N (%)** | **hrHPV^+^, N (%)** | ***P* ^b^** |  |
| **PD-L1s** | 178 |  |  | 0.661 |  |
| Negative | 84 (47.2) | 58 (48.3) | 26 (44.8) |  |  |
| Positive | 94 (52.8) | 62 (51.7) | 32 (55.2) |  |  |
| **PD-L1t** | 178 |  |  | 0.156 |  |
| Negative | 58 (32.6) | 42 (35.0) | 16 (27.6) |  |  |
| Marginal | 80 (44.9) | 48 (40.0) | 32 (55.2) |  |  |
| Diffuse | 40 (22.5) | 30 (25.0) | 10 (17.2 |  |  |
| **Siglec15s** | 170 |  |  | 0.274 |  |
| Negative | 14 (8.2) | 11 (9.6) | 3 (5.4) |  |  |
| Low，1-50 | 130 (76.5) | 83 (72.8) | 47 (83.9) |  |  |
| High，≥50 | 26 (15.3) | 20 (17.5) | 6 (10.7) |  |  |
| **Siglect15t** | 170 |  |  | 0.758 |  |
| Negative | 61 (35.9) | 40 (35.1) | 21 (37.5) |  |  |
| Positive | 109 (64.1) | 74 (64.9) | 35 (62.5) |  |  |

^a^ t-test, ^b^ Chi-square test; hrHPV^-^: high-risk human papillomavirus negative; hrHPV^+^: high-risk human papillomavirus positive; The suffix s represents the stromal region, and the suffix t represents the intratumoral region. Significant values (*P* < 0.05) are indicated as bold.

**Table S2.** Univariable and multivariate COX regression analysis for disease-specific survival (DSS) based on the optimal cut-point variables of immune markers.

| **Variable** | **Contrast** | **Univariable analysis** | |  | **Multivariate analysis** | |
| --- | --- | --- | --- | --- | --- | --- |
|  |  | **HR [95% CI]** | ***P*** |  | **HR [95% CI]** | ***P*** |
| **Tumor-microenvironmental parameters** | | | | | | |
| **hrHPV status** | Positive vs. Negative | 0.414[0.192-0.891] | **0.024** |  | 0.367[0.165-0.818] | **0.014** |
| PD-L1 pattern | Positive vs. Negative | 0.744[0.406-1.366] | 0.340 |  |  |  |
|  | Marginal vs. negative | 0.470[0.244-0.983] | **0.045** |  | - | - |
|  | Diffuse vs. marginal | 3.055[1.423-6.557] | **0.004** |  | - | - |
|  | Diffuse vs. Negative/marginal | 2.067[1.106-3.864] | **0.023** |  | 2.382[1.112-5.099] | **0.025** |
| PD-L1s | Positive vs. Negative | 0.590[0.325-1.072] | **0.084** |  | - | - |
| CD8s | High vs. Low | 0.503[0.248-1.019] | **0.056** |  | - | - |
| CD8t | High vs. Low | 0.799[0.338-1.889] | 0.609 |  |  |  |
| FOXP3s | High vs. Low | 0.382[0.209-0.697] | **0.002** |  | 0.366[0.180-5.099] | **0.006** |
| FOXP3t | High vs. Low | 0.423[0.196-0.911] | **0.028** |  | - | - |
| GrBs | High vs. Low | 0.497[0.274-0.900] | **0.021** |  | - | - |
| GrBt | High vs. Low | 0.313[0.172-0.568] | **<0.001** |  | - | - |
| CD68s | High vs. Low | 0.284[0.150-0.536] | **<0.001** |  | - | - |
| CD68t | High vs. Low | 0.569[0.293-1.106] | **0.096** |  | - | - |
| CD206s | High vs. Low | 0.446[0.244-0.813] | **0.008** |  | - | - |
| CD206t | High vs. Low | 0.349[0.193-0.633] | **0.001** |  | - | - |
| PD-1s | High vs. Low | 0.590[0.325-1.072] | **0.084** |  |  |  |
| PD-1t | High vs. Low | 0.499[0.276-0.901] | **0.021** |  | 0.452[0.236-0.864] | **0.016** |
| CTLA-4s | High vs. Low | 0.367[0.192-0.701] | **0.002** |  | - | - |
| Siglec15s | Negative | 1 | **0.013** |  | - | - |
|  | Low | 0.404[0.187-0.876] | **0.022** |  | - | - |
|  | High | 0.161[0.043-0.606] | **0.007** |  | - | - |
| Siglect15s | Positive vs. Negative | 0.629[0.347-1.139] | 0.126 |  |  |  |
| **Clinicopathological parameters** | | | | | | |
| Age | Per year | 1.017[0.994-1.040] | 0.153 |  |  |  |
| Tumor grade | Per grade | 1.962[1.303-2.955] | **0.001** |  | - | - |
| T stage | Per T stage | 2.416[1.756-3.325] | **<0.001** |  | 1.848[1.206-2.830] | **0.005** |
| N stage | N+ vs N0 | 37.50[9.07-155.15] | **<0.001** |  | 30.001[7.09-123.91] | **<0.001** |
| LVI | Present vs. absent | 40.16[9.708-166.11] | **<0.001** |  | - | - |
| NI | Present vs. absent | 3.449[1.725-6.897] | **<0.001** |  | - | - |
| Necrosis | Present vs. absent | 2.013[0.984-4.115] | 0.055 |  |  |  |
| Histological Subtypes | Related vs. Non-related | 0.205[0.028-1.488] | 0.117 |  |  |  |

The suffix s represents the stromal region, and the suffix t represents the intratumoral region; LVI: lymphovascular invasion; NI: nerve invasion; Bold indicates significant difference.

**Table S3.** Univariable and multivariate COX regression analysis for disease-specific survival (DSS) based on the continuous variables of immune markers.

| **Variable** | **Contrast** | **Univariable analysis** | |  | **Multivariate analysis** | |
| --- | --- | --- | --- | --- | --- | --- |
|  |  | **HR [95% CI]** | ***P*** |  | **HR [95% CI]** | ***P*** |
| **Tumor-microenvironmental parameters** | | | | | | |
| **hrHPV status** | Positive vs. Negative | 0.414[0.192-0.891] | **0.024** |  | 0.343[0.152-0.771] | **0.010** |
| PD-L1 pattern | Positive vs. Negative | 0.744[0.406-1.366] | 0.340 |  |  |  |
|  | Marginal vs. negative | 0.470[0.244-0.983] | **0.045** |  | - | - |
|  | Diffuse vs. marginal | 3.055[1.423-6.557] | **0.004** |  | - | - |
|  | Diffuse vs. Negative/marginal | 2.067[1.106-3.864] | **0.023** |  | 1.864[0.904-3.844] | 0.092 |
| PD-L1s | Positive vs. Negative | 0.590[0.325-1.072] | **0.084** |  | - | - |
| CD8s | Per cell/mm2 | 0.924[0.769-1.110] | 0.397 |  |  |  |
| CD8t | Per cell/mm2 | 0.903[0.759-1.074] | 0.248 |  |  |  |
| FOXP3s | Per cell/mm2 | 0.670[0.542-0.828] | **<0.001** |  | 0.737[0.580-0.937] | **0.013** |
| FOXP3t | Per cell/mm2 | 0.861[0.685-1.082] | 0.199 |  |  |  |
| GrBs | Per cell/mm2 | 0.704[0.531-0.933] | **0.015** |  | - | - |
| GrBt | Per cell/mm2 | 0.650[0.505-0.836] | **0.001** |  | - | - |
| CD68s | Per cell/mm2 | 0.867[0.665-1.130] | 0.289 |  |  |  |
| CD68t | Per cell/mm2 | 0.992[0.693-1.421] | 0.967 |  |  |  |
| CD206s | Per cell/mm2 | 0.852[0.701-1.035] | 0.106 |  |  |  |
| CD206t | Per cell/mm2 | 0.761[0.618-0.937] | **0.010** |  | 0.806[0.643-1.011] | 0.062 |
| PD-1s | Per cell/mm2 | 0.847[0.685-1.049] | 0.127 |  |  |  |
| PD-1t | Per cell/mm2 | 0.768[0.574-1.029] | **0.077** |  | - | **-** |
| CTLA-4s | Per cell/mm2 | 0.734[0.574-0.938] | **0.014** |  | - | - |
| Siglec15s | Per score | 0.742[0.576-0.956] | **0.021** |  | - | - |
| Siglect15t | Per score | 0.806[0.599-1.085] | 0.155 |  |  |  |
| **Clinicopathological parameters** | | | | | | |
| Age | Per year | 1.017[0.994-1.040] | 0.153 |  |  |  |
| Tumor grade | Per grade | 1.962[1.303-2.955] | **0.001** |  | - | - |
| T stage | Per T stage | 2.416[1.756-3.325] | **<0.001** |  | 1.758[1.112-2.780] | **0.016** |
| N stage | N+ vs N0 | 37.50[9.07-155.15] | **<0.001** |  | 32.778[7.73-139.09] | **<0.001** |
| LVI | Present vs. absent | 40.16[9.708-166.11] | **<0.001** |  | - | - |
| NI | Present vs. absent | 3.449[1.725-6.897] | **<0.001** |  | - | - |
| Necrosis | Present vs. absent | 2.013[0.984-4.115] | 0.055 |  |  |  |
| Histological Subtypes | Related vs. Non-related | 0.205[0.028-1.488] | 0.117 |  |  |  |

The suffix s represents the stromal region, and the suffix t represents the intratumoral region; LVI: lymphovascular invasion; NI: nerve invasion; Bold indicates significant difference.

**Table S4.** Univariable and multivariate logistic regression analysis of clinicopathological factors associated with lymph node metastasis (LNM).

| **Marker** | **Contrast** | **Univariable analysis** | |  | **Multivariate analysis** | |
| --- | --- | --- | --- | --- | --- | --- |
|  |  | **OR [95% CI]** | ***P*** |  | **OR [95% CI]** | ***P*** |
| **Tumor-microenvironmental parameters** | | | | | | |
| **hrHPV status** | Positive vs. Negative | 1.069[0.553-2.067] | 0.843 |  |  |  |
| PD-L1 pattern | Positive vs. Negative | 1.047[0.555-1.977] | 0.887 |  |  |  |
|  | Marginal vs. negative | 0.896[0.450-1.786] | 0.896 |  |  |  |
|  | Diffuse vs. marginal | 1.518[0.735-3.400] | 0.241 |  |  |  |
|  | Diffuse vs. Negative/marginal | 1.509[0.744-3.061] | 0.254 |  |  |  |
| PD-L1s | Positive vs. Negative | 0.943[0.666-1.633] | 0.738 |  |  |  |
| CD8s | Per cell/mm2 | 0.852 [0.685-1.059] | 0.149 |  |  |  |
| CD8t | Per cell/mm2 | 0.974 [0.818-1.160] | 0.767 |  |  |  |
| FOXP3s | Per cell/mm2 | 0.071[0.789-1.021] | **0.071** |  | - | - |
| FOXP3t | Per cell/mm2 | 0.964[0.763-1.219] | 0.761 |  |  |  |
| GrBs | Per cell/mm2 | 0.732[0.526-1.020] | **0.066** |  | - | - |
| GrBt | Per cell/mm2 | 0.576[0.416-0.796] | **0.001** |  | 0.577[0.376-0.885] | **0.012** |
| CD68s | Per cell/mm2 | 0.358[0.147-0.874] | **0.024** |  | - | - |
| CD68t | Per cell/mm2 | 0.915[0.625-1.338] | 0.646 |  |  |  |
| CD206s | Per cell/mm2 | 0.908 [0.733-1.125] | 0.378 |  |  |  |
| CD206t | Per cell/mm2 | 0.823 [0.667-1.015] | **0.069** |  | - | - |
| PD-1s | Per cell/mm2 | 0.178 [0.694-1.070] | 0.178 |  |  |  |
| PD-1t | Per cell/mm2 | 0.963[0.736-1.260] | 0.783 |  |  |  |
| CTLA-4s | Per cell/mm2 | 0.735[0.571-0.945] | **0.016** |  | 0.659[0.469-0.926] | **0.016** |
| Siglec15s | Per score | 0.866[0.664-1.129] | 0.287 |  |  |  |
| Siglect15t | Per score | 1.005[0.757-1.335] | 0.970 |  |  |  |
| **Clinicopathological parameters** | | | | | | |
| Age | Per year | 0.991[0.969-1.014] | 0.427 |  |  |  |
| Tumor grade | Per grade | 3.172[1.867-5.388] | **＜0.001** |  | 3.392[1.956-7.866] | **＜0.001** |
| T stage | Per T stage | 2.625[1.662-4.146] | **＜0.001** |  | 1.708[0.940-3.102] | 0.079 |
| LVI | Present vs. absent | 37.627[4.898-289.052] | **＜0.001** |  | 41.370[2.55-671.64] | **0.009** |
| NI | Present vs. absent | 2.951[1.260-6.912] | **0.013** |  | - | - |
| Necrosis | Present vs. absent | 3.095[1.167-8.212 | **0.023** |  | - | - |
| Histological Subtypes | Related vs. Non-related | 0.302[0.083-1.102] | **0.070** |  | 0.152[0.026-0.891] | **0.037** |

The suffix s represents the stromal region, and the suffix t represents the intratumoral region; LVI: lymphovascular invasion; NI: nerve invasion; Bold indicates significant difference.

**Table S5.** PD-L1 and Siglec-15 associated with immune clusters.

| **Variable** | **Total, N (%)** | **Cluster A, N (%)** | **Cluster B, N (%)** | **Cluster C, N (%)** | **Cluster D, N (%)** | ***P*** |
| --- | --- | --- | --- | --- | --- | --- |
| **PD-L1s** |  |  |  |  |  | **< 0.001** |
| Negative | 76 (46.6) | 30_a_ (100.0) | 2_b_ (8.3) | 10_b, c_ (27.0) | 34_c_ (41.5) |  |
| Positive | 87 (53.4) | 0_a_ (0.0) | 22_b_ (91.7) | 17_b, c_ (63.0) | 48_c_ (58.5) |  |
| **PD-L1t** |  |  |  |  |  | **< 0.001** |
| Negative | 50 (30.7) | 22_a_ (73.3) | 1_b_ (4.1) | 7_b_ (25.9) | 20_b_ (24.4) |  |
| Marginal | 75 (46.0) | 6_a_ (20.0) | 10_a, b_ (41.7) | 12_a, b_ (44.4) | 47_b_ (57.3) |  |
| Diffuse | 38 (23.3) | 2_a_ (6.7) | 12_b_ (54.2) | 8_a, b_ (29.6) | 13_a_ (18.3) |  |
| **Siglec-15s** |  |  |  |  |  | **< 0.001** |
| Negative | 12 (8.0) | 8_a_ (26.7) | 1_a, b_ (4.2) | 3_a, b_ (11.1) | 1_b_ (1.2) |  |
| Low，1-50 | 126 (77.3) | 22_a_ (83.3) | 20_a_ (83.3) | 19_a_ (70.4) | 65_a_ (79.3) |  |
| High，≥50 | 24 (14.7) | 0_a_ (0.0) | 3_a_ (12.5) | 5_a_ (18.5) | 16_a_ (19.5) |  |
| **Siglect-15t** |  |  |  |  |  | **< 0.001** |
| Negative | 57 (35.0) | 20_a_ (66.7) | 4_b_ (16.7) | 11_a, b_ (40.7) | 22_b_ (26.8) |  |
| Positive | 106 (65.0) | 10_a_ (33.3) | 20_b_ (83.3) | 16_a, b_ (59.3) | 60_b_ (73.2) |  |

The suffix s represents the stromal region, and the suffix t represents the intratumoral region. Bold indicates significant difference. Bonferroni correction. Each subscript letter (a, b or c) denotes a subset of mutation categories whose column proportions do not differ significantly from each other at the 0.05 level.

**Table S6.** Role of immune markers in tumor progression and the represented inflammatory cell types.

| **Variable** | **Level of expression** | **Role in progression** | **Class of inflammatory cells** |
| --- | --- | --- | --- |
| CD8 | high | inhibition | Cytotoxic T cell |
| FOXP3 | high | promotion | Regulatory T cell |
| GrB | high | inhibition | Cytotoxic T cell granules |
| CD68 | high | inhibition | Macrophage |
| CD206 | high | promotion | M2 macrophage |
| PD-1 | high | promotion | Immune checkpoint |
| CTLA-4 | high | promotion | Immune checkpoint |
| PD-L1 | high | promotion | Immune checkpoint |
| Siglec15 | high | promotion | Immune checkpoint |

**Table S7.** Levels of immune markers expressed in different clusters A-D.

| **Variable** | **Cluster A** | **Cluster B** | **Cluster C** | **Cluster D** |
| --- | --- | --- | --- | --- |
| CD8s | ↓ | ↑ | → | → |
| CD8t | ↓ | ↑ | ↓ | ↓ |
| FOXP3s | ↓ | ↑ | → | → |
| FOXP3t | ↓ | ↑ | → | → |
| GrBs | ↓ | ↑ | → | → |
| GrBt | ↓ | ↑ | → | → |
| CD68s | ↓ | ↑ | → | → |
| CD68t | ↓ | ↑ | → | → |
| CD206s | ↓ | ↑ | ↓ | → |
| CD206t | ↓ | ↑ | ↓ | → |
| PD-1s | ↓ | ↑ | → | → |
| PD-1t | ↓ | ↑ | → | → |
| CTLA-4s | → | → | → | → |
| PD-L1s | ↓ | ↑ | → | → |
| PD-L1t | ↓ | ↑ | → | → |
| Siglec15s | → | → | → | → |
| Siglec15t | ↓ | ↑ | → | ↑ |

The suffix s represents the stromal region, and the suffix t represents the intratumoral region. The high expression of immunomarker is indicated by up arrow (↑), and low expression is indicated by down arrow (↓). Horizontal arrow (→) indicate moderate level.


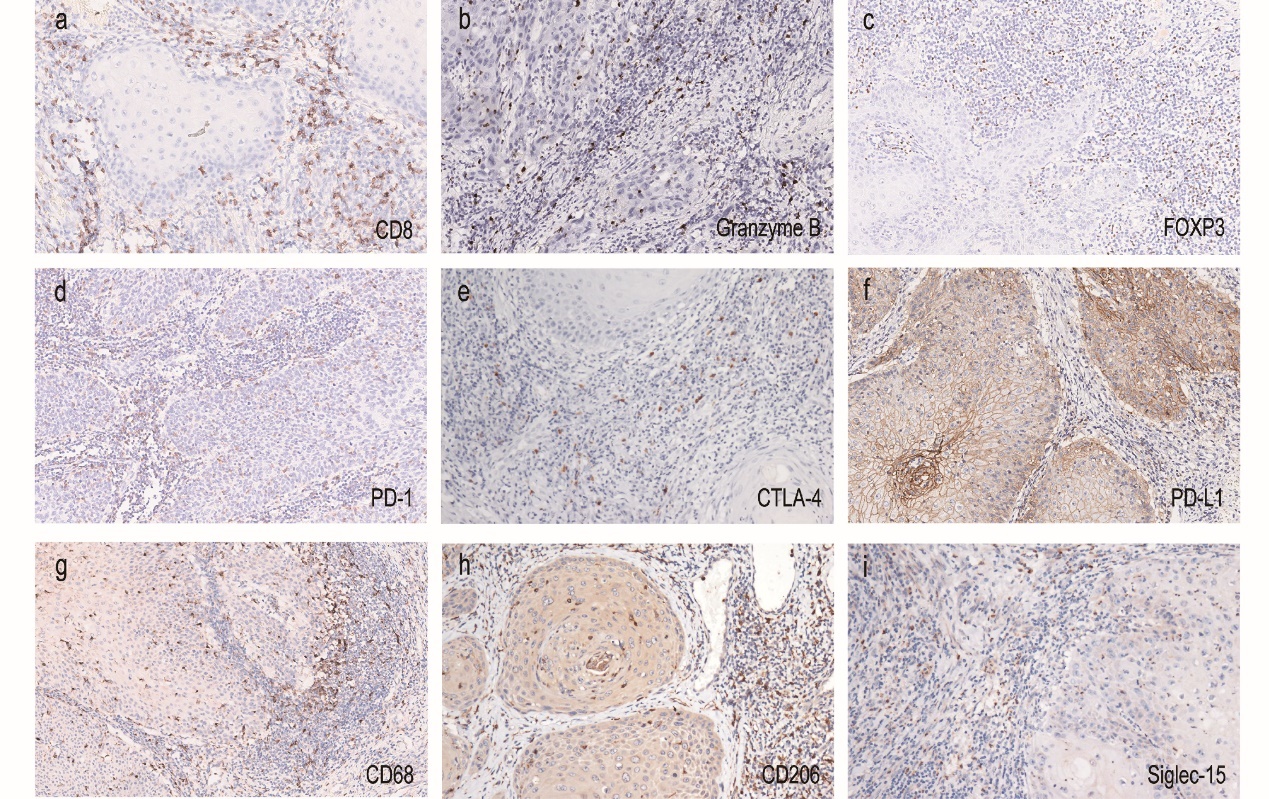


**Figure S1.** Representative figures on IHC staining for 9 immune markers. (**a**) Strong CD8 membrane and cytoplasm staining. (**b**) Strong granzyme B cytoplasm staining. (**c**) Strong FOXP3 nucleus staining. (**d**) PD-1 cytoplasm and membrane staining. (**e**) CTLA-4 membrane and cytoplasm staining in stroma. (**f**) PD-L1 membrane staining in tumor. (**g**) Strong CD68 and (**h**) strong CD206 cytoplasm staining in stroma tumor. (**i**) Siglec-15 cytoplasm staining in stroma. Magnification: 200×.


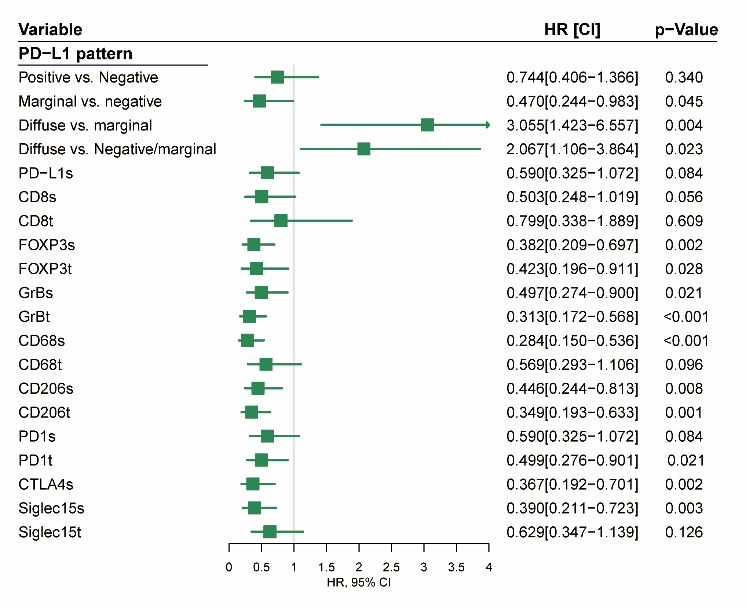


**Figure S2.** Forest plot of hazard ratios (HR) for disease-specific survival (DSS). For statistical analysis, the densities of immune markers were treated as categorical variables. The suffix s represents the stromal region, and the suffix t represents the intratumoral region.


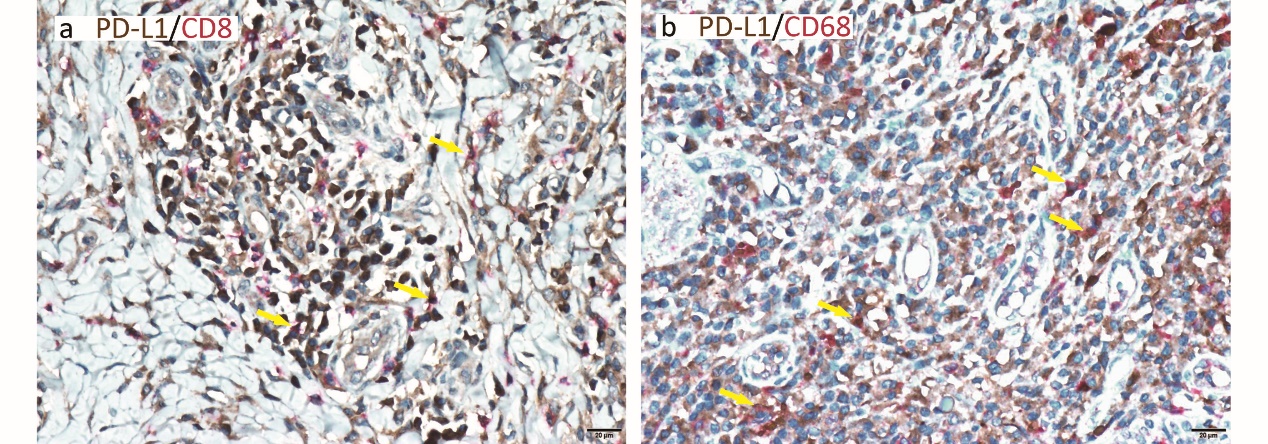


**Figure S3.** Representative images of dual IHC staining for PD-L1/CD8(a) and PD-L1/CD68(b). Arrowheads indicate double-stained cells. Magnification: 400×.


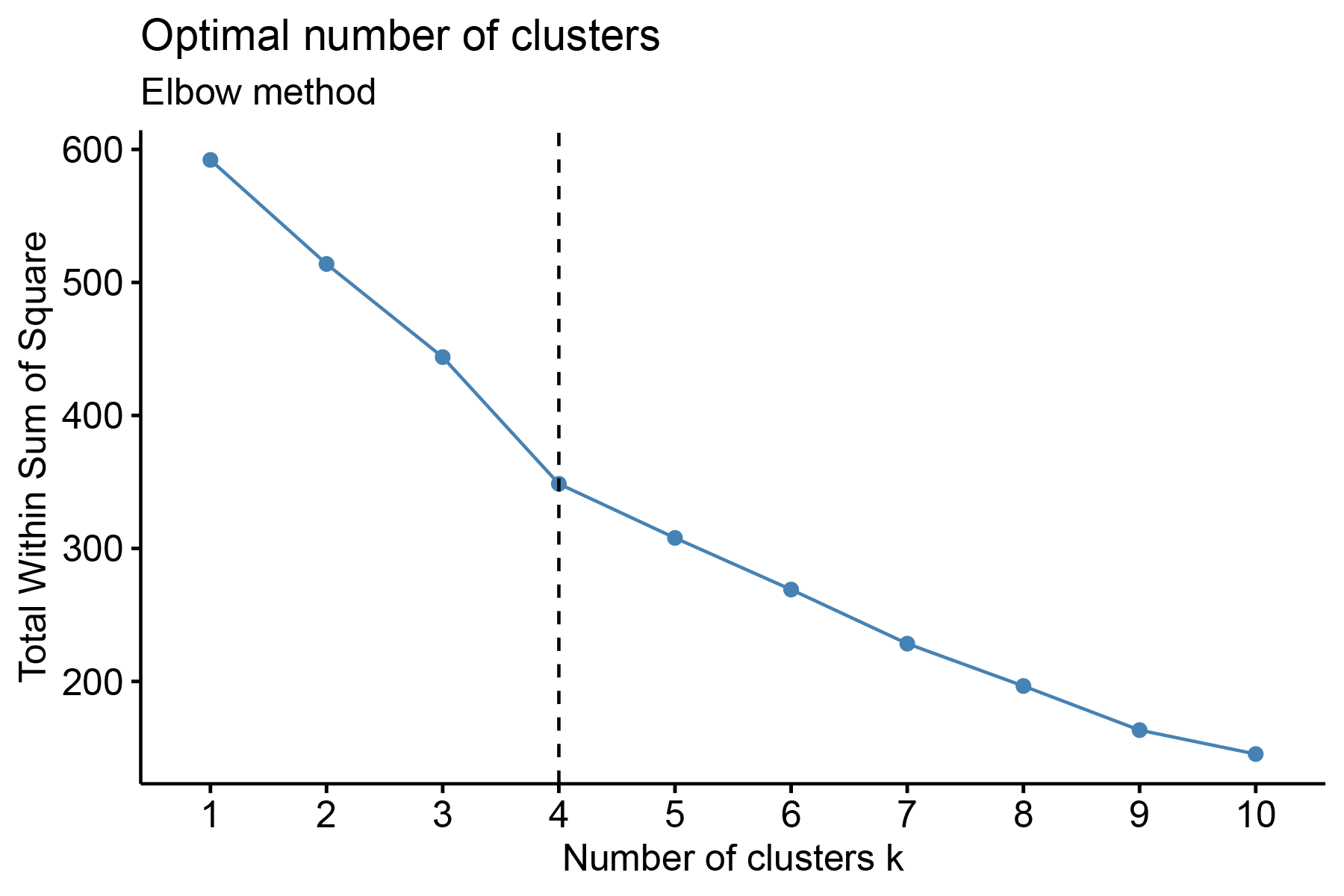


**Figure S4.** Elbow plot shows that four is the optimal number of clusters as it appears to be the bend in the elbow. Elbow plot was made by using “factoextra” R package.


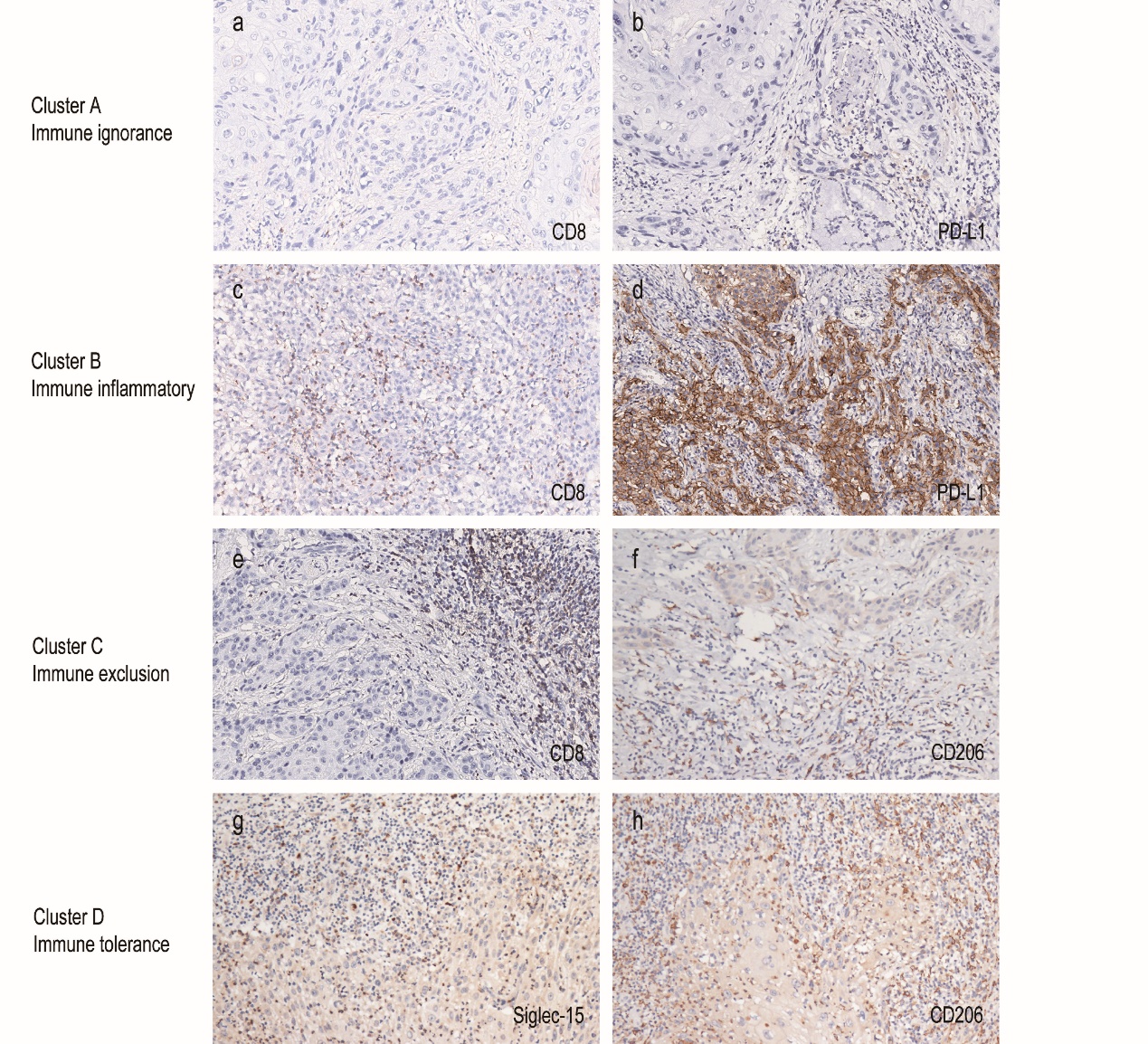


**Figure S5.** Representative IHC images of immune markers in different immunophenotypes. Negative expression of CD8 (**a**) and PD-L1 (**b**) in cluster A (immune ignorance). High expression of CD8 in stroma and tumor (**c**) and diffuse intratumoral PD-L1 expression (**d**) in cluster B (immune inflammatory). High stromal CD8 (**e**) and CD206 (**f**) expression but low intratumoral CD8 and CD206 expression in cluster C (immune exclusion). High expression of Siglec-15 (**g**) and CD206 (**h**) in stroma and tumor in cluster D (immune tolerance). Magnification: 200×.
